# The Kager Triangle: An Anatomic Review and Potential Implications for Lymphatic Surgery

**DOI:** 10.1097/GOX.0000000000006791

**Published:** 2025-05-05

**Authors:** Yuma Fuse, James E. Fanning, Travis Boyd, Meera Singhal, Jordan Bohnen, Fernando Raduan, Dhruv Singhal

**Affiliations:** From the * Division of Plastic and Reconstructive Surgery, Department of Surgery, Beth Israel Deaconess Medical Center, Harvard Medical School, Boston, MA; † Department of Plastic Surgery, University of Texas Southwestern, Dallas, TX; ‡ Lewis Katz School of Medicine, Temple University, Philadelphia, PA; § Department of Surgery, Beth Israel Deaconess Medical Center, Harvard Medical School, Boston, MA; ¶ Department of Orthopedic Surgery, Beth Israel Deaconess Medical Center, Harvard Medical School, Boston, MA.

## Abstract

Vascularized lymph node transplant (VLNT) is widely performed for chronic upper and lower extremity lymphedema. However, ideal recipient sites for the transplant are still under debate. The placement of VLNTs distally in an extremity can be challenging as the small cross-sectional area of the limb at this level does not allow for flap inset without gross contour deformity, which can adversely impact aesthetic outcomes and preclude fitting of an adequate compression garment. In this article, we introduce the Kager triangle as a potential distal lower extremity VLNT recipient site for the lower extremity. The Kager triangle is bordered by the Achilles tendon, the flexor hallucis longus, and the calcaneus, which accommodates the Kager fat pad, the largest adipose structure in the lower extremity. We transferred an omentum lymph node flap to the Kager triangle, and the posterior tibial artery and the anterior lateral malleolar artery were utilized as recipient vessels in a flow-through fashion. The incisions were directly closed with excellent cosmesis.

**Takeaways**

**Question:** How can the challenge of placing a vascularized lymph node transplant in the distal lower extremity be overcome given that the limb’s small cross-sectional area does not allow for flap inset without gross contour deformity?

**Findings:** The Kager fat pad was replaced with an omental lymph node flap. The posterior tibial and anterior lateral malleolar arteries were utilized as recipient vessels in a flow-through fashion. The incisions were directly closed with excellent cosmesis.

**Meaning:** The Kager triangle represents an ideal vascularized lymph node transplant recipient site, enabling primary closure of incisions with favorable aesthetic outcomes that allow for the use of standard compression garments.

## INTRODUCTION

Free vascularized lymph node transplant (VLNT) is widely performed for chronic extremity lymphedema, yielding reliable results. Although some surgeons prefer proximal placement to clear the scar and bridge the lymphatic gap created by prior lymphadenectomy, others prefer distal placement because fluid predominantly accumulates in the distal extremity.^1^ In lower extremity lymphedema, transplantation to the ankle may provide the most favorable outcomes.

Disadvantages of distal placement include a smaller space for composite transfer, resulting in poor aesthetic outcomes and inadequate compression garment fitting.^1^ The senior surgeon (D.S.) initially anastomosed distal VLNTs to the anterior tibial vessels but abandoned this approach due to poor cosmetic outcomes and transitioned to VLNTs to the medial sural vessels. With the goal of returning to the distal extremity while preserving cosmesis and adequate compression, we introduce the Kager triangle as a VLNT recipient site.

The role of the Kager triangle, which contains a fat pad, remains unclear. Histological studies of the Kager triangle suggest proprioceptive function, as the fat pad has a high concentration of mechanoreceptors. However, ankle ligaments (lateral, medial, and syndesmotic),^2^ the ankle capsule, tendons, muscles,^3^ and even ankle skin also express these receptors, which can compensate for the absence of the Kager fat pad.^4^ An additional function of the Kager fat pad may be the prevention of adhesions by keeping the Achilles tendon and the other flexor tendons such as the flexor hallucis longus, flexor digitorum longus, and the posterior tibial tendon apart.^5^ Notably, posterior ankle approaches to treat Achilles tendon tendinopathies and posterior malleolar fractures involve resection of the Kager fat pad and have been performed for decades without complications, such as adhesions.

### The Anatomy of the Kager Triangle

The Kager fat pad is one of the largest adipose structures in the leg, with a volume of 10.6 mL.^6^ The fat pad is bordered by the flexor hallucis longus, Achilles tendon, and calcaneus,^5^ and functions as a fat spacer, providing a mechanical advantage to the ankle. The Achilles-associated part protects blood vessels running to the tendon, the flexor hallucis longus–associated part helps the bursal wedge during plantar flexion, and the bursal wedge minimizes pressure changes in the retrocalcaneal bursa.^7,8^

The blood supply to the Kager triangle is provided by posterior tibial and peroneal arteries.^5^ Medially, posterior tibial vessels and the tibial nerve are located posterior to the medial malleolus. Laterally, the peroneal artery divides into posterior and anterior branches. The posterior branch further branches toward the lateral malleolus, anastomosing with the anterior lateral malleolar artery from the anterior tibial artery. The ankle’s vasculature is variable, and the peroneal artery becomes dominant when the posterior tibial artery is absent.

Of note, at the level of the Achilles tendon body, the blood supply of the tendon does not come from the Kager fat pad but from the anterior paratenon, entering the tendon transversely and traveling along the fibers.^8^ To the best of our knowledge, vessels traveling from the Kager fat pad to the Achilles tendon have not been described.

## CASE REPORT

**Fig. 1.**
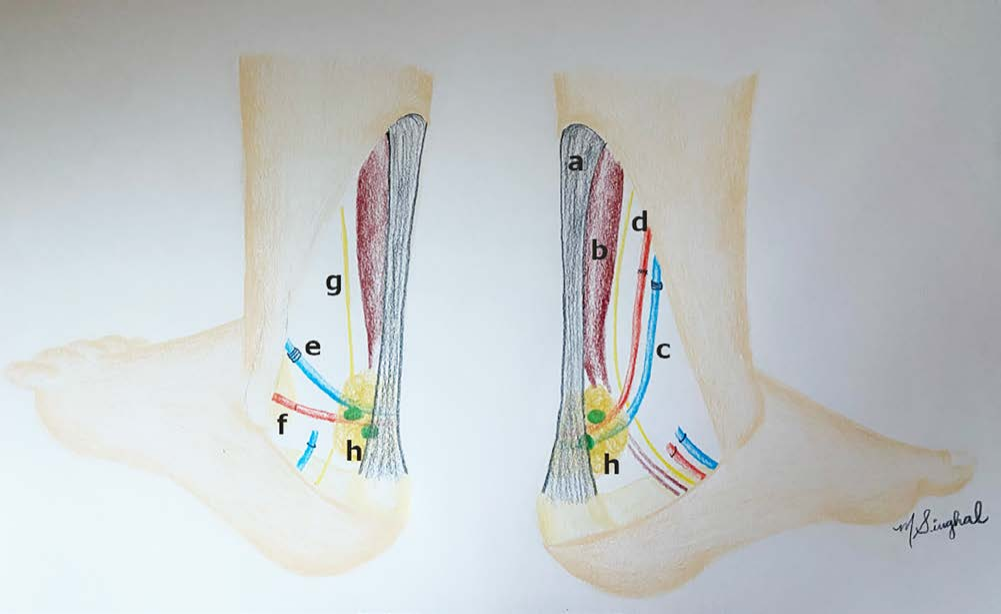
Flap inset. An omentum LN flap was transplanted into the Kager triangle. The posterior tibial artery with its vena comitans and the anterior lateral malleolar artery with the lessor saphenous vein were utilized as recipient vessels. a, Achilles tendon; b, flexor hallucis longus; c, posterior tibial; d, tibial nerve; e, lessor saphenous vein; f, anterior lateral malleolar artery; g, sural nerve; h, omental flap.

A 36-year-old woman developed chronic left lower extremity lymphedema secondary to cervical cancer treated with pelvic lymph node (LN) dissection. Lymphedema was managed conservatively with compression and then with power-assisted liposuction 3 years prior. After liposuction, she converted from fat-dominant to fluid-dominant lymphedema. Upon evaluation with indocyanine green lymphography, no appropriate linear channels for lymphovenous bypass were identified. Omental VLNT was offered to improve residual symptoms centered at the ankle and reduce compression requirements. We performed a double-level transfer, anastomosing our first transplant to the medial sural vessels. Working with our foot and ankle team, we replaced the Kager fat pad with the omental VLNT as our second transfer. The second transfer is described in Figure 1.

**Fig. 2.**
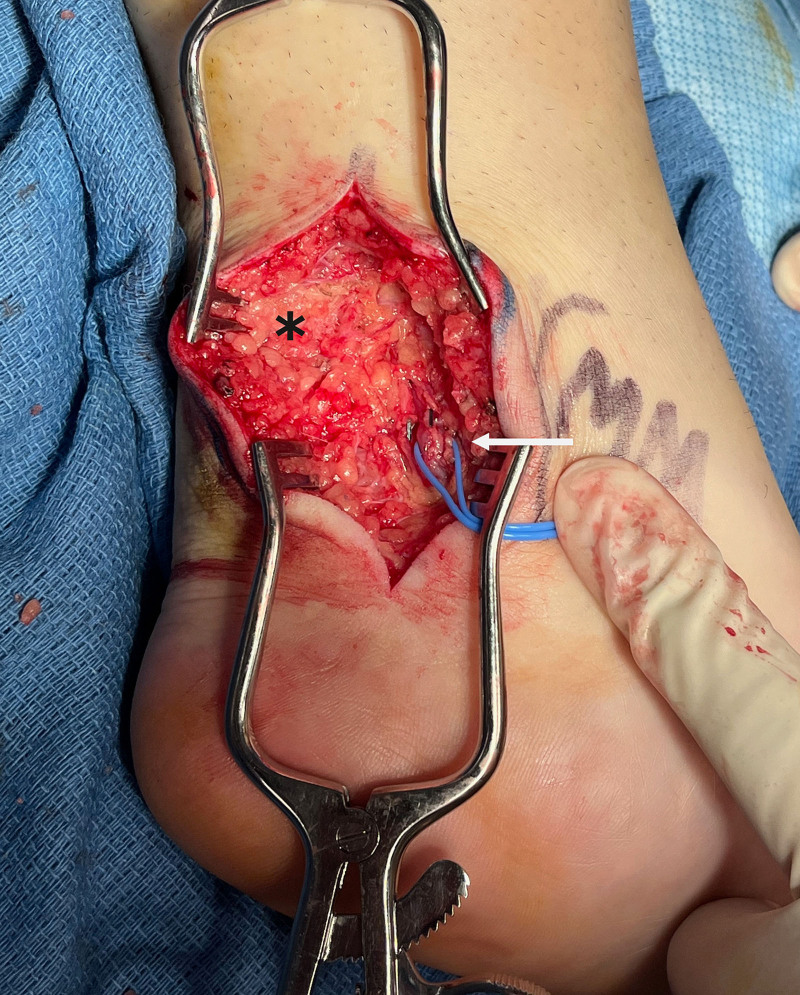
Kager fat (*) was exposed through the medial incision. The posterior tibial vessels (white arrow) were isolated with the tibial nerve.

Posterior tibial and anterior lateral malleolar vessels were marked preoperatively with ultrasound. In the supine position, a longitudinal incision was made posterior to the medial malleolus, the retinaculum was opened, and posterior tibial vessels were exposed and prepared. The tibial nerve was identified and preserved. With the Kager triangle exposed (Fig. 2), the fat pad was excised, paying careful attention to maintain the paratenon over the Achilles tendon. The incision was temporarily closed, and the patient was repositioned prone. A longitudinal incision was made posterior to the lateral malleolus. The anterior lateral malleolar artery, adjacent vein, and sural nerve were identified and isolated. The lateral aspect of the Kager triangle was visualized, and the remaining contents were excised (total weight: 6 g).

**Fig. 3.**
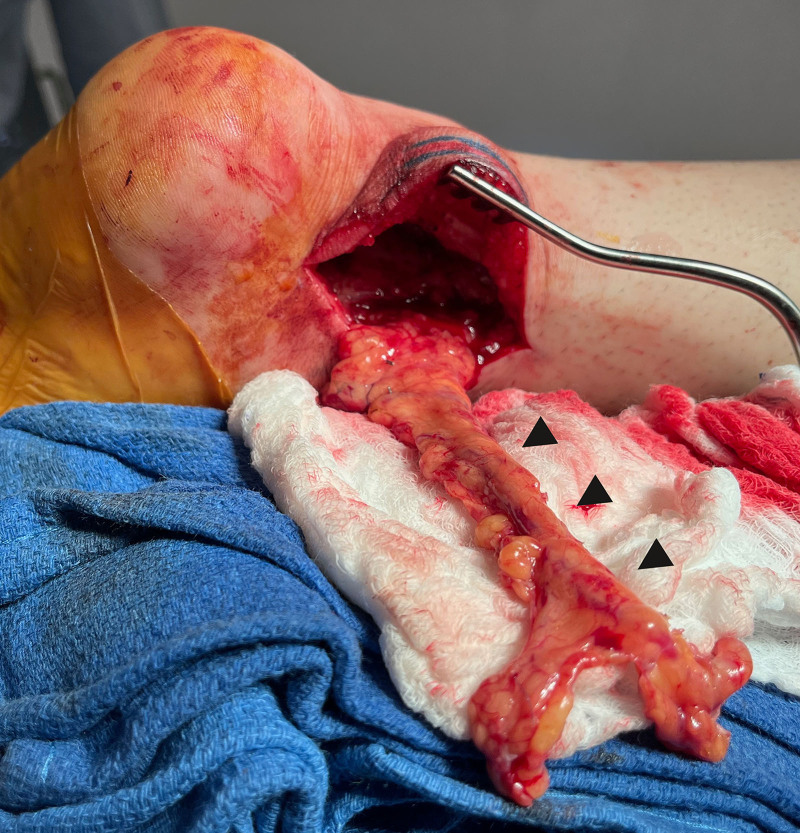
Flap inset. The right gastroepiploic vessels were anastomosed to the posterior tibial vessels before the flap (black arrowheads) was placed through the Kager triangle.

The patient was repositioned supine. The omental VLNT was laparoscopically harvested by the general surgery team and contained 7 LNs visualized with intraoperative ultrasound. The patient was then repositioned prone. The flap was divided, with the proximal part containing 5 LNs transplanted to the popliteal region as previously reported. The distal part containing 2 LNs and weighing 10 g was transplanted to the Kager triangle. First, proximal vessels were anastomosed to posterior tibial vessels in an end-to-end fashion, and then the flap was reflected through the Kager triangle (Fig. 3). Next, distal vessels were anastomosed to anterior lateral malleolar vessels in an end-to-end fashion, thereby completing the flow-through configuration. Finally, incisions were closed directly over Penrose drains.

**Fig. 4.**
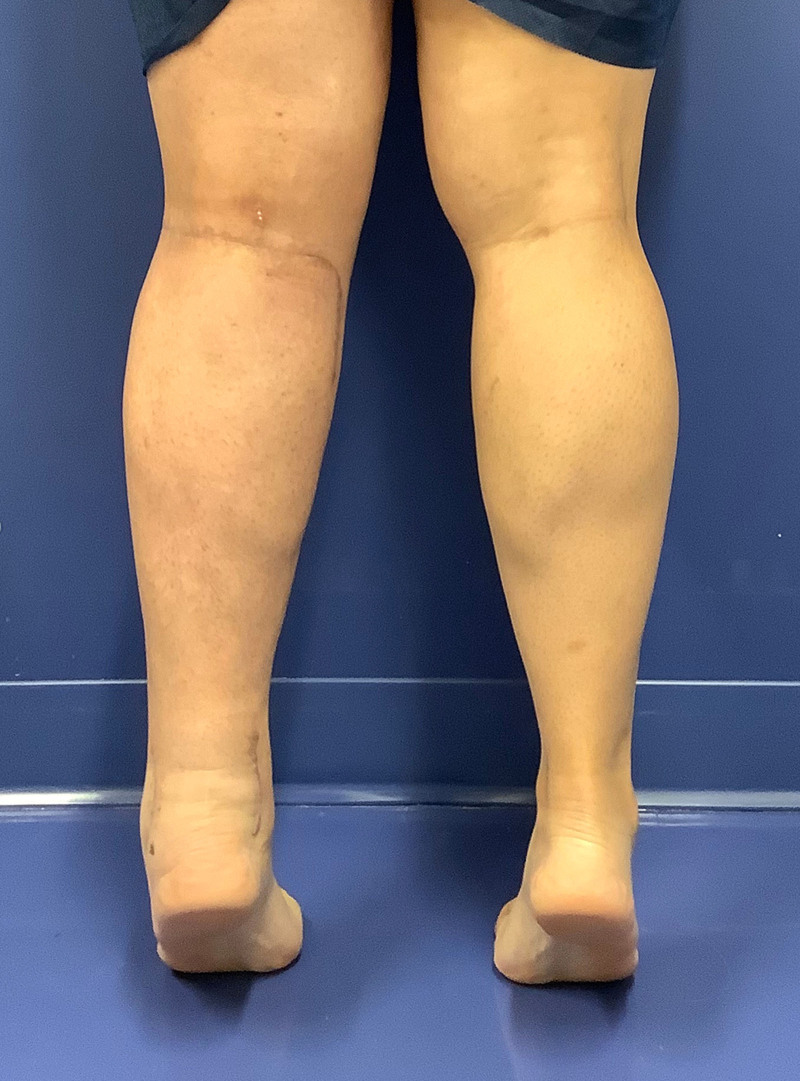
Postoperative view at 3-month follow-up.

A multi-podus boot was applied to the distal leg to avoid compression of the VLNT and immobilize the foot postoperatively. The next day, patency of the anastomoses was confirmed by a radiology attending with color Doppler ultrasound. The patient resumed compression garments at the 3-week follow-up. Postoperatively, the patient demonstrates intact sensory and motor function of the sural and tibial nerves and normal ankle motion (Fig. 4). (See Video [online], which displays the postoperative view at 3 months of follow-up and shows no limitation of plantar flexion or extension. The replacement of the Kager fat pad with omentum prevents contact between the Achilles and the other tendons, thus avoiding adhesions.) Her relative limb volume difference improved from 0.6% preoperatively to −3.6% at 6 months, whereas her L-Dex score decreased from 52.3 to 36.6.

**Video 1:** which displays the postoperative view at 3 months of follow-up and shows no limitation of plantar flexion or extension. The replacement of the Kager fat pad with omentum prevents contact between the Achilles and the other tendons, thus avoiding adhesions.

## DISCUSSION

As extremity lymphedema disproportionately affects the distal limb, distal VLNTs hypothetically allow for optimal “pump” function^1^ while leveraging gravity. Replacement of the Kager fat pad with a VLNT enables primary closure of the incisions, achieving favorable aesthetic outcomes and standard compression garment fitting.

An additional benefit of the Kager triangle as a VLNT recipient site may include connectivity to the deep lymphatic system. Barbieux et al^9^ visualized the lower extremity deep system on lymphoscintigraphy with radiocolloid tracer injection into the Kager triangle. VLNTs in the Kager triangle would theoretically establish connections between the superficial and deep lymphatic systems via lymphangiogenesis, facilitating fluid pumping into the deep system. The effectiveness of placement in the Kager triangle may be explained by both pumping and lymphangiogenesis theories.

The ankle’s vascular network is variable. If the lateral tibial artery was not present or inadequate as a recipient vessel, consideration would be given to exposing the main trunk of the peroneal artery with a distal osteotomy of the fibula. Preoperative computed tomography angiography may be considered to evaluate the anatomy.

A potential drawback of this procedure is the need for patient repositioning and the involvement of multiple surgical teams. We recently modified repositioning to a single instance. Specifically, the ankle is prepared through both medial and lateral incisions in the supine position while the flap is harvested. The patient is then repositioned to the prone position for preparation of the popliteal area.

The Kager triangle presents an ideal anatomical recipient site for lower extremity VLNT distal placement. Advantages include sufficient volume to accommodate the flap and potential bridging between superficial and deep lymphatic systems. There is no evidence of adverse sequelae on ankle function.

## DISCLOSURES

The authors have no financial interest to declare in relation to the content of this article. This study was supported, in part, by the National Heart, Lung, and Blood Institute of the National Institutes of Health under award number R01HL157991 (Dr. Singhal) and the NIH Common Fund under award number U54HL165440 (Dr. Singhal). Fanning is supported by the 2024 JOBST Lymphatic Research Grant awarded by the Boston Lymphatic Symposium, Inc.
